# Yerba Santa (Eriodyction Glutinosium)

**Published:** 1877-07-20

**Authors:** 

**Affiliations:** Aurora, Ill.


### YERBA SANTA (ERIODYCTION GLUTINOSIUM.)

By Dr. GABEL, Aurora, Ill.

This agent was first introduced to the profession by Dr. Bundy, of Colusa, Cal., and I can well recall the thoughts which ran through my mind when I first read his report in the *Eclectic Medical Journal* of several cases treated with the remedy. I thought it another sensation from some over enthusiastic person. But as I had several patients who were, and had been for some time, troubled with bronchitis of an atonic nature, and I had no direct remedy at my command, it was an easy matter for me to reason that all of our best remedies were once new; and thus I concluded to send to Dr. B. for some of the "Yerba Santa," which I received in due time; made some into a saturated tincture of all alcohol, and some into a tincture of alcohol 70, and after using both I found the latter tincture the better of the two, both being given at different times to the same patient. I give the leading symptoms of one case, in order to point out the specification of the drug.

Mrs. M., a lady about forty-five years old, came to me for consultation. She had been treated by physicians of all schools, in this city as well as Chicago. She did not expect to be helped by any one, for the cough which was troubling her did not abate under all the array of medical skill; and she was tired taking medicine, and was satisfied she had confirmed consumption, for some of her physicians had told her so. Upon examination I found that this cough she had had for five years, and got worse whether she took medicine or not. She was greatly emaciated, limbs inclined to be dropsical, digestive apparatus out of order, as it always is in cases of this kind, pulse weak and 96, quite nervous, bronchial tubes dilated, blowing sounds very distinct all over the chest, also a moist rattling sound showing an accumulation of mucus in the smaller bronchial tubes, respiration about 26, tongue broad, relaxed and pale. All of the symptoms were such as to discourage me, and I hesitated to tell her what she wanted to know, viz., if I thought I could help her. I told her that if she would take medicine for two weeks, I could tell her if I could help her. This she consented to do. Prescribed—R. Tinc. Yerba Santa oz. j, glycerine oz. iij. M. Sig., a teaspoonful four times a day. In one week she returned. Upon examination found her some better; renewed the prescription, and in another week she herself said that she would run the risk of my curing her. Cough less, expectorates easy, blowing sounds much better, pulse 85, respiration 22, digestion greatly improved; and right here let me say, that the remedy is an excellent anti-dyspeptic in atonic conditions of this kind. To make a long story short, this lady took the above prescription for two months, and was well; now ten months since, and she is as well as she has ever been.

Miss N., a teacher, came with cough and hemoptysis. Found bronchial tubes dilated, and in an atonic condition. Prescribed—R. Tinc. Yerba Santa oz. ss, glycerine oz. iiss. M. S., a teaspoonful three or four times a day—when all the symptoms disappeared.

Mr. H., atonic bronchitis helped in a very short time. Mr. M., atonic bronchitis, cough for years, weak and much emaciated, digestion very bad. R. Tinc. Yerba Santa oz. j, glycerine oz. iij. M. In about five weeks discharged cured.

Mrs. P., bronchitis with abscess of left lung—usual symptoms. R. Tinc. Yerba Santa oz. j, glycerine oz. iij. M. S., a teaspoonful every three hours. After she had taken this for two days she raised about a pint of yellow-greenish matter; felt quite easy about the chest. Gave the above three times. Lung troubles all removed. Gave glycerine and muriated tincture of iron, for about three or four weeks. This is nearly a year since, and the lady, although old, feels well; no more cough, and what surprises me is that she has no more trouble with the abscess.

The point I wish to make is, that this agent is a remedy in *atonic* conditions only; in inflammation it is worse than useless.—*Eclectic Medical Journal.*

